# Impact of COVID-19 pandemic on the management of patients with RA: a survey of rheumatologists in six European countries

**DOI:** 10.1093/rap/rkac108

**Published:** 2022-12-13

**Authors:** Pedro M Machado, Patrick Verschueren, Rebecca Grainger, Hannah Jones, James Piercy, Katrien van Beneden, Roberto Caporali, Christian Dejaco, Bruno Fautrel

**Affiliations:** Centre for Rheumatology and Department of Neuromuscular Diseases, University College London, London, UK; Rheumatology Department, KU Leuven, Leuven, Belgium; Department of Medicine, University of Otago, Dunedin, New Zealand; Autoimmune Franchise, Adelphi Real World, Bollington, UK; Health Economics and Outcomes Research, Adelphi Real World, Bollington, UK; Medical Affairs, Galapagos NV, Mechelen, Belgium; Department of Clinical Sciences and Community Health, University of Milan, Milan, Italy; Division of Clinical Rheumatology, ASST Pini-CTO, Milan, Italy; Rheumatology, Medical University of Graz, Graz, Austria; Service de Rhumatologie, Sorbonne Université-Assistance Publique-Hôpitaux de Paris, Paris, France

**Keywords:** RA, attitude of health-care professionals, medical education, quality of health care, health policies, COVID-19, pandemic response

## Abstract

**Objective:**

We aimed to describe, from the perspective of rheumatologists in Europe, how the coronavirus disease 2019 (COVID-19) pandemic has impacted their management of people with RA and the continuing medical education of physicians.

**Methods:**

Rheumatologists participating in the Adelphi RA Disease Specific Programme^TM^ in six European countries were contacted in August and September 2020 for a telephone survey. Rheumatologists were asked seven attitudinal questions on changes to patient management, prescription behaviour and continuing education owing to COVID-19. Results were summarized with descriptive statistics.

**Results:**

The telephone survey was completed by 284 rheumatologists. The most commonly reported changes to patient management were increased utilization of video/telephone consultations (66.5% of respondents), fewer visits (58.5%) and limiting physical contact (58.1%). Furthermore, 67.9% of rheumatologists who indicated that prescribing behaviour had changed switched their patients to self-administered medication, and 60.7% reported not starting patients on targeted synthetic DMARDs, biologic originator DMARDs or biosimilar DMARDs. In total, 57.6% of rheumatologists believed that changes in management would persist. Rheumatologists reported that 38.0% of patients expressed concerns about how COVID-19 would impact treatment, including access to treatment and the risk of infection. The biggest impact on rheumatologist education was a switch to online training and conferences.

**Conclusion:**

All countries saw changes in patient management and prescribing behaviour, including the rapid uptake of telemedicine. It is important that the international rheumatology community learns from these experiences to prepare better for future pandemics and to address ongoing rheumatologist shortages.

Key messages

- The COVID-19 pandemic led to rheumatologists conducting more consultations online or via telephone.
- Rheumatologists reported delaying advanced treatments, switching patients to other treatments or discontinuing advanced treatments.
- Concerns of patients and physicians probably drove changes to prescription behaviour during the pandemic.

## Introduction

RA is a complex long-term condition that requires regular monitoring to inform treatment decisions and balance disease control with patient preferences, side-effects and co-morbidities [1, 2]. The coronavirus disease 2019 (COVID-19) pandemic led to challenges for the management of patients with RA. Public health initiatives forced rheumatologists to cancel or postpone in-person appointments and rapidly take on remote consultations and monitoring (telemedicine) [1, 3, 4]. As updated EULAR recommendations [5] came late in the first wave of the pandemic, rheumatologists had to adapt quickly and flexibly to a changing situation, often with minimal instruction.

Telemedicine is not new. Before the pandemic, telemedicine was a possible method of improving access to rheumatology care and addressing global and regional shortages of rheumatologists [6–8]. Although studies are sparse [7, 9], they indicate that video consultations are accurate, providing a valuable alternative to face-to-face visits when diagnosing, monitoring and following up patients with RA, and are generally well received [7, 9–11]. Historically, uptake of telemedicine has been low owing to difficulties in accessing technology, patients missing face-to-face contact with health-care providers, patients fearing that they might miss important clinical information, and reimbursement issues [10–14]. Significant differences between countries in telemedicine regulation and data protection also pose barriers to uptake [12, 15].

As the pandemic saw a rapid global shift to telemedicine to comply with regional public health guidelines, physicians and legislators had to find ways to overcome the previous barriers. There is now a unique opportunity to learn from these experiences and identify factors that might still need to be addressed to sustain adoption of telemedicine. Furthermore, understanding how telemedicine negatively affects patient care will inform for whom and when telemedicine might be most suitable. This will allow rheumatologists to plan a better response in future pandemics, while informing future studies into telemedicine uptake and efficacy. Considering the global shortage of rheumatologists [6, 16], it is vital that we learn how COVID-19 has impacted access to ongoing physician education and how this might influence future training.

The objectives of this real-world investigation were to leverage the existing cohort of rheumatologists participating in the Adelphi RA Disease Specific Programme^TM^ (DSP) to investigate the perspective of European rheumatologists on the impact of the COVID-19 pandemic on management of their patients with RA, their prescribing behaviour and their continuing medical education.

## Methods

The Adelphi RA DSP was a large, point-in-time survey of physicians and their consulting patients presenting in a real-world clinical setting, which was conducted in Belgium, France, Germany, Italy, Spain and the UK between November 2019 and November 2020. The DSP provided a cohort of physicians to conduct a dedicated survey on how COVID-19 impacted patient management and the prescribing behaviour of physicians. The overall DSP methodology has been published previously [17].

The DSP protocol used to collect the original sample and follow-up interview fulfil the definition of market research as defined by European Pharmaceutical Market Research Association (EphMRA) guidance [18] and are therefore exempt from independent review board/clinical research ethics committee review. To confirm this status, the DSP methodology was submitted to the Western Independent Review Board, who provided a letter of exemption (protocol #AG-8382). Permission to contact physicians for a follow-up survey was provided during the initial DSP data-collection period. The follow-up survey was also classified as market research under EphMRA guidance.

Rheumatologists were re-contacted during August and September 2020 for an additional telephone survey comprising attitudinal questions regarding how COVID-19 impacted their management of patients with RA. Participation in the follow-up interview was voluntary; no personally identifiable or protected data were collected, and all data collected were anonymized. Participating physicians received the equivalent of £20 compensation for their time. The telephone survey asked seven questions, grouped into four themes around changes to clinical practice owing to COVID-19: patient management, prescription behaviour, continuing medical education and patient concerns. The term ‘advanced therapy’ was used to refer collectively to biologic or targeted synthetic DMARDs. The telephone survey questions and mode of answer are provided in Supplementary Table S1, available at *Rheumatology Advances in Practice* online.

For quantitative data, the number of respondents for each answer were aggregated for all countries and analysed using descriptive statistics. Results were interpreted at an overall European level. In addition, country-specific analyses were carried out, as appropriate. Thematic analysis [19] was carried out on free-text comments from question 6, which asked rheumatologists to describe patient concerns.

## Results

Of the 316 rheumatologists who participated in the Adelphi RA DSP, 96% (*n* = 284) completed the supplemental COVID-19 survey. Country breakdown and demographics are presented in Supplementary Table S2, available at *Rheumatology Advances in Practice* online.

### Changes to clinical practice owing to COVID-19

#### Effect of COVID-19 on current patient management for RA

Almost all (*n* = 282, 99.3%) rheumatologists reported that COVID-19 had impacted patient management (Table 1). The most common changes reported were ‘moving to video/telephone consultation and remote completion of questionnaires’ (*n* = 189; 66.5%), ‘fewer visits made by individual patients (reduced visiting schedule)’ (*n* = 166; 58.5%), ‘limiting physical contact during consultations (e.g. blood tests)’ (*n* = 165; 58.1%) and ‘fewer visits made by individual patients (postponing visits instigated by patients)’ (*n* = 161; 56.7%).

**Table 1.** Percentage of physicians in each country who selected each response option in response to the question, ‘How has COVID-19 impacted your patient management for RA?’

	Base	Belgium	France	Germany	Italy	Spain	UK
	(*n* = 284)	(*n* = 10)	(*n* = 50)	(*n* = 58)	(*n* = 59)	(*n* = 57)	(*n* = 50)
	
Response	Frequency of response selection (% physicians)						
Moving to video/telephone consultation, moving to remote completion of questionnaires	66.5	90.0	70.0	36.2	47.5	86.0	94.0
Fewer visits made by individual patients (reduced visiting schedule)	58.5	90.0	80.0	–	40.7	96.5	76.0
Limiting physical contact during consultations (e.g. blood tests)	58.1	50.0	42.0	100.0	37.3	57.9	52.0
Fewer visits made by individual patients (postponing visits instigated by patients)	56.7	80.0	70.0	17.2	62.7	64.9	68.0
Only allowing more severe patients (i.e. you/your practice cancelling routine appointments with mild patients)	46.1	20.0	54.0	–	72.9	47.4	64.0
Fewer new patients referred from primary care	43.7	80.0	70.0	–	40.7	54.4	52.0
Fewer tests/investigations performed^a^	38.0	40.0	50.0	–	42.4	38.6	64.0
Changed the way I choose and prescribe medication	29.6	20.0	24.0	13.8	23.7	57.9	30.0
Moving to remote completion of questionnaires (yes or no answer)	5.6	–	8.0	–	8.5	5.3	8.0
Other (specify)	1.8	–	–	–	6.8	–	2.0
COVID-19 has not impacted patient management	0.7	–	2.0	–	1.7	–	–

a Overlap between limiting physical contact and fewer tests/investigations is possible.

COVID-19: coronavirus disease 2019.

Several differences were noted between countries (Table 1). Moving to video/telephone consultations was less common in Germany (*n* = 21 of 58; 36.2%), whereas 86.0% (*n* = 49 of 57) of rheumatologists in Spain and 94.0% (*n* = 47 of 50) in the UK indicated that video/telephone consultations were conducted more frequently. Notably, 100.0% of German rheumatologists reported ‘limiting physical contact during consultations (e.g. blood tests)’, whereas 37.3% (*n* = 22 of 59) of Italian rheumatologists reported the same. Prioritizing patients with severe disease over routine appointments was reported by 46.1% of rheumatologists in Europe (from 0.0% in Germany to 72.9% in Italy). In addition, 29.6% (*n* = 84) of rheumatologists indicated that they changed how they chose and prescribed medication.

#### Effect of COVID-19 on prescribing behaviour

Overall, 67.9% (*n* = 57) of the 84 rheumatologists who indicated that the COVID-19 pandemic changed their prescribing behaviour reported that they ‘changed medication to self-administration’, with variation from 57.6% (*n* = 19 of 33) in Spain to 100.0% (*n* = 15 of 15) in the UK. More than half of the rheumatologists reported ‘not starting new patients on an advanced therapy treatment’ (*n* = 51; 60.7%), except in Germany, where rheumatologists reported that COVID-19 had not affected advanced therapy prescribing behaviour. When considering patients already on advanced treatments, 60.0% of rheumatologists in the UK, 27.3% in Spain and 8.3% in France reported switching therapy class; 46.7%, 21.2% and 25.0%, respectively, lowered the dose; and 40.0% and 15.2% halted advanced therapy altogether in Spain and the UK (Table 2).

**Table 2.** Percentage of physicians in each country who selected each response option in response to the question, ‘How has COVID-19 impacted the way you prescribe medicine?’

	Base	Belgium	France	Germany	Italy	Spain	UK
	(*n* = 84)	(*n* = 2)	(*n* = 12)	(*n* = 8)	(*n* = 14)	(*n* = 33)	(*n* = 15)
	
Response	Frequency of response selection (% physicians)						
Changed medication to self-administration	67.9	–	58.3	87.5	64.3	57.6	100.0
Not starting new patients on an advanced therapy treatment	60.7	100.0	58.3	–	28.6	87.9	60.0
Changed medication to a treatment with less frequent dose	32.1	–	16.7	62.5	7.1	33.3	53.3
Prescribed a longer course of treatment	31.0	–	25.0	–	35.7	36.4	40.0
Changed advanced therapy (biologic or JAK inhibitor treatment) to different advanced therapy (biologic or JAK inhibitor treatment)	28.6	–	16.7	–	–	42.4	53.3
Halted advanced therapy treatment, switched to different treatment class	22.6	–	8.3	–	–	27.3	60.0
Kept current medication, but reduced frequency of dosing	20.2	–	25.0	–	–	21.2	46.7
Halted advanced therapy treatment, no replacement treatment prescribed	13.1	–	–	–	–	15.2	40.0
Other (specify)	6.0	–	8.3	–	–	3.0	20.0

COVID-19: coronavirus disease 2019; JAK: Janus kinase.

#### Effect of COVID-19 on future patient management and prescribing behaviour for RA

**Figure 1.**
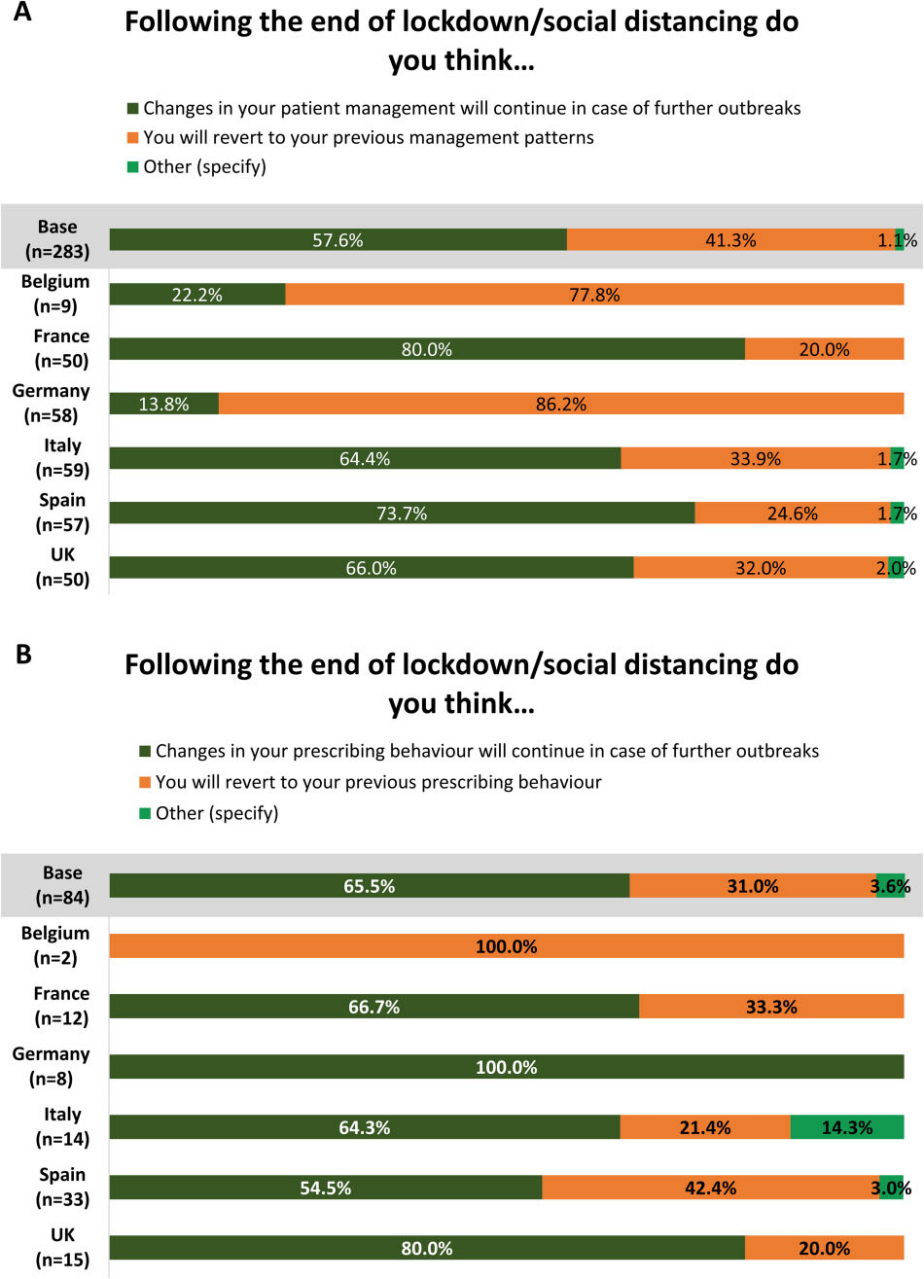
Proportion of physicians who selected each response to the following question regarding the likelihood of (**A**) changes to patient management and (**B**) changes to prescribing behaviour continuing after the end of lockdown

When asked whether they thought changes in patient management and prescribing behaviour would continue after the end of lockdown/social distancing, 57.6% (*n* = 163) of rheumatologists reported that changes would continue in the event of further outbreaks. Furthermore, 65.5% (*n* = 55) of the 84 rheumatologists who reported changes to prescribing behaviour believed that these would continue in the event of future COVID-19 outbreaks. In Germany and Belgium, however, most rheumatologists (*n* = 50, 86.2%, and *n* = 7, 77.8%, respectively) stated that they would revert to previous management patterns after the end of lockdown/social distancing. If Germany and Belgium were excluded, 70.8% (153 of 216) of physicians believed that management changes would continue (Fig. 1).

#### Patient concerns about COVID-19 impact

**Figure 2.**
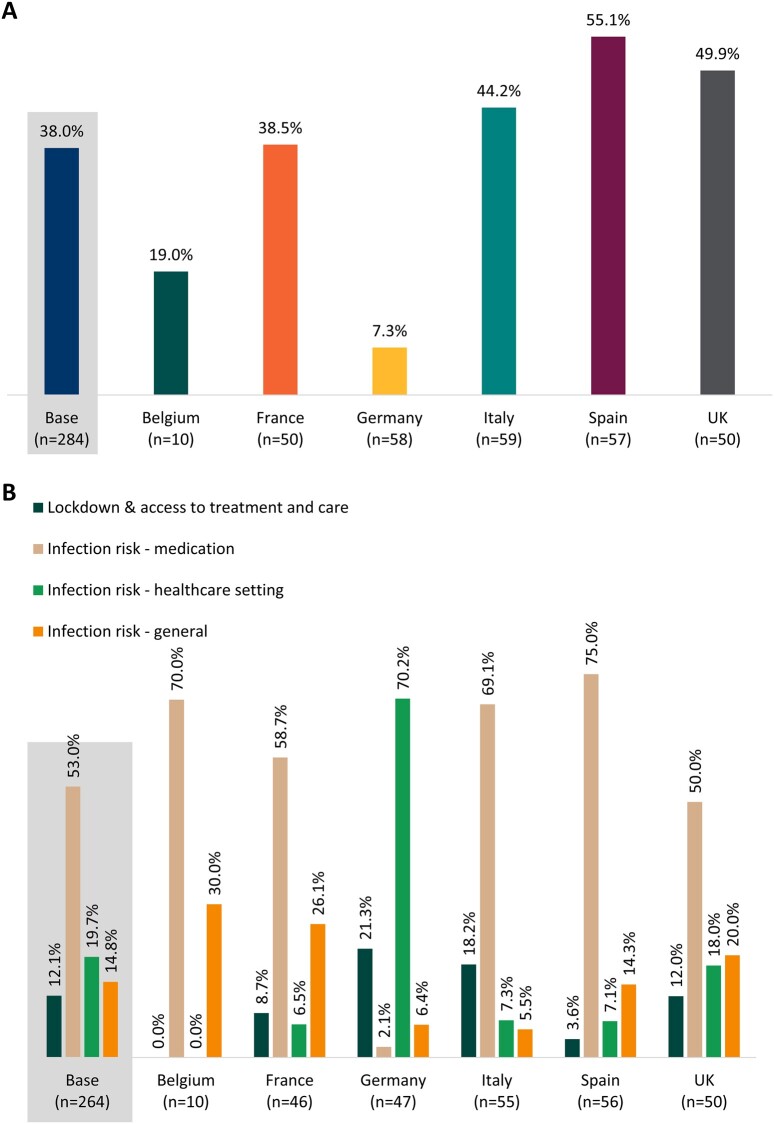
Patient concerns for each country, as reported by their physicians. (**A**) Proportion of patients who expressed concerns owing to coronavirus disease 2019 (COVID-19) about their treatment regimen as estimated by rheumatologists. (**B**) The percentage of rheumatologists who mentioned each of the four major themes identified by thematic analysis when asked to describe patient concerns

Rheumatologists estimated that, on average, 38.0% of patients had expressed concerns about their treatment regimen owing to COVID-19, ranging from 7.3% in Germany to 55.1% in Spain (Fig. 2A). Four concern themes were identified: ‘lockdown and access to treatment and care’; ‘infection risk—medication’ (e.g. increased risk of COVID-19 owing to immunosuppression or method of medicine administration); ‘infection risk—health-care setting’ (e.g. risk of infection from attending hospital); and ‘infection risk—general’ (e.g. infection risk from having to leave the house or travel).

Thematic analysis showed that the most frequent patient concern reported by rheumatologists was infection risk owing to medication, which was reported by 140 of 264 (53.0%) rheumatologists. This was also the case in all individual countries, except Germany, where the main patient concern (70.2%, 33 of 47) was the risk of contracting COVID-19 from a health-care setting (Fig. 2).

#### Effect of COVID-19 on continuing medical education

**Figure 3.**
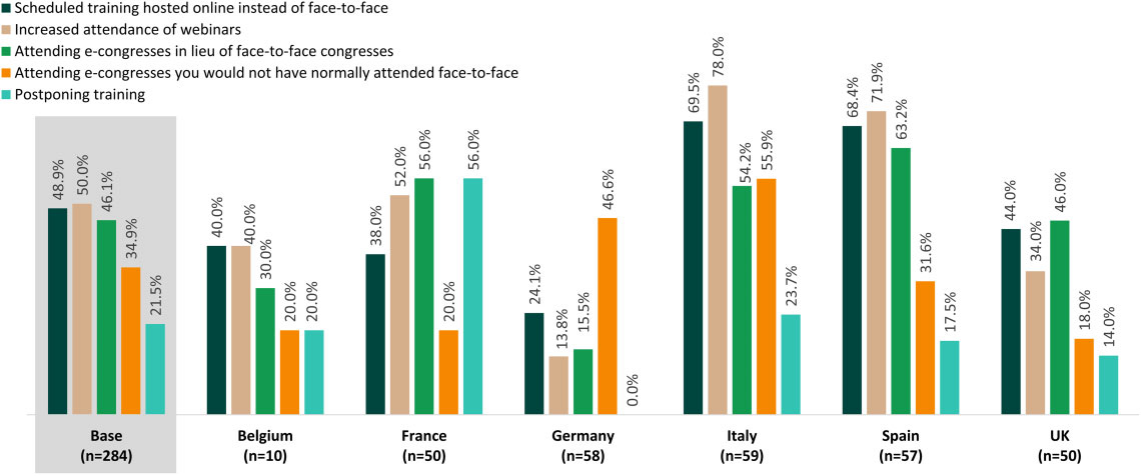
Changes to continuing education owing to the coronavirus disease 2019 (COVID-19) pandemic and the proportion of physicians who reported them

Finally, when asked how COVID-19 had affected continuing medical education, most rheumatologists reported some changes (Fig. 3). Half (*n* = 142 of 284) reported increased attendance of webinars, 48.9% (*n* = 139) reported scheduled training hosted online instead of face to face, and 46.1% (*n* = 131) reported attending e-congresses in lieu of face-to-face congresses. These rates were higher in Italy and Spain. Furthermore, around half of rheumatologists in Germany and Italy (46.6%, *n* = 27 of 58, and 55.9%, *n* = 33 of 59, respectively) reported attending e-congresses that they would not normally have attended face to face (Fig. 3).

## Discussion

Overall, rheumatologists across all countries made changes to the way in which they managed patients with RA and their prescribing behaviour; most believed that these changes would continue in the event of future outbreaks. There was significant variation between countries in the nature and extent of these changes, with Germany showing fewer adaptations compared with other countries. Furthermore, rheumatologists in all countries undertook ongoing medical education and conferences online. Here, we consider these findings in the context of pandemic responses in each country to understand their implications and produce future guidance for rheumatology practice.

### Patient management decisions during the COVID-19 pandemic

Rheumatologists across all countries surveyed saw increased use of telemedicine during the pandemic. Most reported some reduction in the frequency of appointments, prioritizing in-person appointments for severe patients and reducing new referrals from primary care. These findings were reflected in a recent survey of 1286 health-care professionals in rheumatology [20], wherein 82% indicated cancellation/postponement of in-person appointments for new patients, with 84% offering remote consultation. Furthermore, 91% of physicians cancelled/postponed follow-up visits, with 96% offering remote follow-ups [20]. Only Germany reported no changes to patient priority or referral numbers, with only minimal changes to visit frequency reported. The main change made to patient management in Germany was to decrease physical contact.

At the time of our survey, countries had different infection rates and public health responses. All countries showed an increase in COVID-19 cases from August to September 2020, but only France, Spain and the UK saw high case numbers, with Italy, Germany and Belgium keeping daily case numbers <2000. At survey initiation, only Germany, France and the UK were not in a lockdown. The UK had strong recommendations to stay home in place, and France was already seeing a rapid increase in COVID-19 cases, which might explain why Germany reported fewer changes in patient contact than other countries [21]. This is potentially supported by our finding that German physicians believed that management changes would not persist, because changes might have begun to revert by the time of our survey. Surveys in Latin America and the UK that showed similar telemedicine utilization during the pandemic to our study showed that lower proportions of rheumatologists believed that telemedicine would continue [22, 23]. In particular, a UK study conducted during August–December 2020 showed that the majority of rheumatologists believed that <50% of follow-up appointments post-pandemic would be conducted remotely; although still high, this could potentially indicate a cooling of opinions about telemedicine after the initial pandemic peak [22]. These differences might also indicate that, in our survey, rheumatologists believed other changes besides remote consultation might continue (i.e. decreased physical contact or wearing face masks).

We must also consider differences in telemedicine regulations and remuneration between countries [15]. Only several months into the pandemic did legislation begin to change in countries to allow greater uptake of telemedicine. The countries that reported the lowest levels of switching to telemedicine were also those with the lowest remuneration for teleconsultations: German rheumatologists received no teleconsultation remuneration until legislation in April 2020 [24]; in Belgium, rheumatologists received only a small remuneration of €20 [25]; and in Italy, remuneration was limited to a small number of specific services [26]. A survey of rheumatologists in the Middle East and North Africa showed that, for all regions, only 12% of telemedicine appointments were reimbursed; in this case, only 54% fully agreed to using telemedicine, with a further 24% saying that they would agree if it is reimbursed [27]. These findings, alongside our own, indicate that if telemedicine is to become part of regular practice, reimbursement must be the same as that for in-person appointments.

In most countries surveyed, patients were seen in public hospitals (Supplementary Table S2, available at *Rheumatology Advances in Practice* online). The COVID-19 pandemic led to many hospitals redeploying staff from chronic disease care to COVID-19 care [22, 28]. Many countries saw closure of rheumatology services owing to staff redeployment, meaning that appointments were cancelled or postponed and/or switched to telemedicine [20, 22]. A perceived higher risk of COVID-19 infection in hospitals might also have led to cancellation of appointments, both by physicians and at the request of patients [29, 30]. Health funding is also likely to have influenced appointment cancellation; rheumatologists who were paid based on the number of consultations would have been disincentivized to cancel appointments because this would have led to reduced income. In summary, the structure and location of health-care delivery and its funding seem likely, and not surprisingly, to have influenced rheumatology care during the pandemic.

There is still inadequate empirical evidence to guide clinical practice via telemedicine in rheumatology. Recent updates to EULAR recommendations for pandemic responses removed several recommendations revolving around telemedicine, in favour of addressing the issue via a recently formed taskforce, indicating that there are still lessons to be learned [31]. The Asia Pacific League of Associations for Rheumatology recently released recommendations highlighting the need to assess the suitability of telemedicine for each patient on a case-by-case basis [32]. Telemedicine might be a mechanism for addressing the global shortage and maldistribution of rheumatologists, and it has already been shown to improve access to rheumatologists for more remote patients in Australia and rural New England [33, 34]. Although it is clear that there is a patient-led demand for telemedicine [8, 16], studies indicate that telemedicine is underutilized by ethnic minorities and patients with lower socioeconomic status, with issues such as literacy, access to new technologies and willingness to embrace them leading to disparities in patient outcome and access to health care [35]. A possible solution to access issues is the approach adopted by the Alaska Native Medical Center, in which patients travel to local clinics, with technology in place for remote consultations and clinicians on hand to assist [14]. It is clear, however, that although telemedicine might be suitable for any patient at any time, it is unlikely to be suitable for all patients, all the time. It is key, therefore, that telemedicine does not replace traditional face-to-face appointments but is integrated on a case-by-case basis and tailored to individual patient needs [36].

### Physician prescribing behaviour during the pandemic

In our survey, rheumatologist-reported changes to prescribing behaviour largely consisted of changing medication to self-administration, avoiding the initiation of advanced therapies and, in some countries, reducing the dose of or discontinuing already prescribed advanced therapies. German rheumatologists, however, did not change advanced therapy prescribing behaviour. A previous study found that treatment decisions were often postponed (34%), and most health-care professionals in rheumatology (74%) stated that it was less likely for patients to start a biological/targeted synthetic DMARD during the first wave of the pandemic, mainly owing to patients’ fear of starting such treatments, limited availability of screening procedures and decreased availability of rheumatological services [20].

In the early stages of the pandemic there were fears that patients with RA were more at risk of infection owing to their condition, particularly for those taking immunosuppressant medication [5]. Physicians and patients feared that immunosuppression could lead to more severe COVID-19 infection, particularly before vaccine development. EULAR recommended that advanced therapy prescriptions be adjusted on a case-by-case basis, considering patient concerns, probably reflecting the experiences of rheumatologists during the first wave [5, 20]. We found that physicians estimated that more than one-third of patients expressed concerns about the impact of COVID-19 on their treatment and that three of the four main concerns revolved around increased infection risk (whether from having to attend a hospital or from their medication). In a UK study, 50% of discontinuations were at the request of patients [22]. Improving patient education and communication, particularly via telemedicine, could be vital in assuaging fears and ensuring that patients maintain optimal treatment in future practice; physicians suggest that unified and consistently applied guidance could help in this [26].

As with patient management, German rheumatologists showed the least change in prescribing behaviour. Unlike other countries in the Adelphi cohort, whose rheumatologists were based in hospitals, German rheumatologists were based in public offices (non-private outpatient practices; Supplementary Table S2, available at *Rheumatology Advances in Practice* online). Staff redeployment was less likely in public offices, and infection risk might have been perceived to be lower by authorities, allowing rheumatologists to maintain in-person appointments. This might have bolstered both physician and patient confidence in the monitoring and safety of advanced therapies, leading to fewer changes. The highest anxiety was reported for infection risk from a health-care setting, consistent with public clinics remaining open. Rheumatologists in Spain and the UK reported the highest levels of patient concern and the highest levels of prescription changes. Moving more rheumatology services to public offices and ensuring that they remain open could be key to relieving hospital burden and ensuring the continuation of treatment in future pandemics.

Several drugs used to treat RA, including tocilizumab, were identified as possible treatments for COVID-19 early in the pandemic [27, 37, 38]. Increased demand for these drugs led to global shortages, meaning that physicians might have been forced to switch treatments [27, 39]. The reduced prescribing of and switching of advanced therapies might have been pragmatic, owing to access issues. Switching could have had the advantage that some biologics have better adherence with self-administration [40–42]. Although self-training for injection via video has generally been well received, rheumatologists might have postponed initiation until in-person training could take place [41]. Even with training, self-injection is not suitable for some patients owing to limited dexterity or patient preference [42–44]. Given that it has been found that delays between symptom onset and initiating DMARDs lead to lower remission rates and worse outcomes in patients [46, 47], ensuring that patients are on the most appropriate RA drug/drugs to begin with and prioritizing access to treatments based on patient needs are important to prevent treatment disruption in future. Diversification of treatment options and working with health authorities to improve supply lines could also be key to maintaining treatment in future pandemics [27].

### Effects of COVID-19 on physician education

In addition to the impacts on patient management and prescribing behaviour, this survey showed that COVID-19 had a marked effect on continuing health-care professional education in all countries, particularly in Italy. Increased attendances at online training, webinars and e-congresses were the three most common changes, with online training consistently ranking within the top three across all countries. Rheumatologists also reported the benefit of increased attendance at conferences that they would not normally have attended. Studies into the effectiveness of more traditional medical education online regularly cite increased accessibility, comfort and a greater ability to meet individual learning needs as major benefits [45, 46]. Conversely, these same studies have shown that online education struggles to find a balance between practical and theoretical learning; communication is often ineffective, and lessons are poorly optimized to the online environment. Furthermore, doctors in training report missing out on face-to-face interaction and networking opportunities and that some balance of online and in-person education would be optimal for future education [4, 45, 47].

It is not only the way education is accessed that has been altered by COVID-19, but also the type of education needed. A recent international report highlighted new training needs in telemedicine, showing that, despite widespread uptake of telemedicine, only 39% of rheumatology trainees received telemedicine training, and many reported feeling less comfortable when evaluating new patients or making treatment changes using telemedicine [4]. If telemedicine is to be incorporated into future models of care, appropriate training in virtual clinical skills will be necessary. Some suggest that this could include simulated virtual consultations and lessons in telemedicine-specific legislation [4]. Indeed, the United States Accreditation Council of Graduate Medical Education has already added telemedicine-specific competencies to its list of core competencies for medical training [4, 14, 48].

### Limitations

Several key limitations need to be considered when interpreting our results. The data presented here are >2 years old and might not reflect current attitudes to telemedicine. Low sample size in some cases means that certain results might not be representative; in such cases (e.g. prescribing behaviour in Belgium) we have presented the data but drawn limited conclusions from those samples. Furthermore, questions on whether changes to practice would continue are based on the opinions of rheumatologists; ultimately, the choice might fall to hospitals and policy-makers rather than rheumatologists. Rheumatologist opinion might influence policy decisions, meaning that our data remain key to understanding the direction that policy might take in future. Despite these limitations, our study reflects a snapshot of rheumatological practice across multiple countries, representing a broad sample of rheumatologists and providing valuable insight into responses to the pandemic and the opinions of rheumatologists at that time.

### Conclusion

Rheumatologists made changes to their prescribing behaviour and the way in which they manage patients, probably to accommodate public health initiatives and to assuage both their own fears and those of patients surrounding medication. We saw differences between countries, owing, in combination, to the pandemic impact and response in each country, treatment setting, and the variability of legislation and remuneration surrounding telemedicine consultations. One key benefit has been the impact on medical education, with opportunities to learn remotely being expanded. Given the potential benefits of remote consultations, it is vital that guidance on telemedicine is harmonized and that issues with reimbursement and patient education around the risks of treatment and remote treatment devices highlighted above are addressed.

## Supplementary Material

rkac108_Supplementary_Data

## Data Availability

All data that support the findings of this study are the intellectual property of Adelphi Real World. All requests for access should be addressed directly to James Piercy at: james.piercy@adelphigroup.com.
