# Supplementary material for: Impact of COVID-19 pandemic on the management of patients with RA: a survey of rheumatologists in six European countries
Source: Rheumatol Adv Pract. 2022 Dec 13;7(1):rkac108. doi: 10.1093/rap/rkac108 (PMC9800854; doi:10.1093/rap/rkac108)
Supplement: rkac108_Supplementary_Data [file rkac108_supplementary_data.pdf]

Supplementary Table S1: Questions included in the RA DSP COVID-19 telephone survey, and mode of answer.

Number	Question	Base	Question type
**1**	How has COVID-19 impacted your patient management for rheumatoid arthritis?	All physicians	Multi-choice
**2**	How has COVID-19 changed the way you prescribe medicine?	All physicians who stated that their prescribing behaviour had changed at question 1	Multi-choice
**3**	Following the end of lockdown/social distancing do you think…<br>1. Changes in your patient management will continue in case of further outbreaks<br>2. You will revert to previous management patterns<br>3. Other (specify)	All physicians	Single choice
**4**	Following the end of lockdown/social distancing do you think…<br>1. Changes in your prescribing will continue in case of further outbreaks<br>2. You will revert to previous prescribing patterns<br>3. Other (specify)	All physicians	Single choice
**5**	What proportion of your patients have expressed concerns due to COVID-19 about their treatment regimen?	All physicians	Answer as an estimated percentage (0-100)
**6**	Please describe their concerns	Physicians who gave an answer of >0% at question 5	Free text – open ended
**7**	How has COVID-19 impacted on the way you undertake medical education?	All physicians	Multi-choice

Supplementary Table S2: Demographics of rheumatologists who completed the supplemental COVID-19 telephone survey.

		Base	Belgium	France	Germany	Italy	Spain	UK
		n=284	n=10	n=50	n=58	n=59	n=57	n=50
Year physician qualified (% physicians)	Before 1983	2.8	-	10.0	-	1.7	-	4.0
	1983-1995	23.9	40.0	42.0	34.5	16.9	19.3	4.0
	1996-2005	30.6	20.0	22.0	51.7	11.9	36.8	32.0
	2006-2016	36.6	30.0	24.0	13.8	52.5	40.4	54.0
	After 2016	6.0	10.0	2.0	-	16.9	3.5	6.0
% patients seen in public hospital	Mean	59.8	65.5	59.0	24.1	66.5	71.6	79.5
	SD	41.4	43.1	41.7	43.2	33.9	30.5	32.9
% patients seen in private hospital	Mean	3.0	10.0	0.7	-	2.5	6.2	4.4
	SD	11.2	23.1	3.0	-	7.4	16.4	14.5
% patients seen in public office	Mean	25.2	0.5	3.4	72.3	19.2	16.3	14.3
	SD	38.0	1.6	17.1	43.7	27.0	24.6	28.3
% patients seen in private office	Mean	12.0	24.0	36.8	3.5	11.7	5.9	1.8
	SD	24.9	36.6	40.2	14.8	18.2	11.3	6.1
% patients seen elsewhere	Mean	-	-	0.1	-	-	-	-
	Min	-	-	-	-	-	-	-

Abbreviations: RA, Rheumatoid Arthritis; SD, Standard Deviation; UK, United Kingdom
